# Knowledge gap in a cross section of Irish general practitioners prescribing denosumab for osteoporosis

**DOI:** 10.1007/s11845-023-03383-w

**Published:** 2023-05-22

**Authors:** Eimear O’ Reilly, Donal Fitzpatrick, Rosaleen Lannon, Kevin McCarroll

**Affiliations:** 1 Royal College of Surgeons, Dublin, Ireland; 2 Bone Health Unit, St James’s Hospital, Dublin, Ireland; 3 Mercer’s Institute for Research on Ageing, Dublin, Ireland

**Keywords:** Denosumab discontinuation, Denosumab knowledge, General practice, Osteoporosis, Rebound bone loss

## Abstract

**Background:**

Denosumab is commonly used by general practitioners (GPs) in Ireland to treat osteoporosis though drug holidays are not recommended with rebound bone loss and risk of vertebral fractures if stopped. We aimed to investigate GP practice and knowledge regarding denosumab including use and reasons for use, therapy duration, blood monitoring and recommended vitamin D status/calcium intake on treatment, staff administering, methods of recall, delays in receiving injections, management of and awarenes of guidelines if stopped, reasons for stopping and concerns about same.

**Methods:**

GPs were contacted (*n* = 846) by email and invited to complete an online anonymous survey comprising 25 questions in January 2022. We collated responses and explored for differences between GP principals/trainers and GP trainees.

**Results:**

There were 146 responses. Sixty-seven percent were female and 50% were GP principal/trainers. Forty-three percent used denosumab as a first line therapy citing convenience in 32% of cases. Half (50%) envisaged therapy for 3–5 years and 15% lifelong use. A fifth (21%) had no concerns about it being stopped (11% trainors vs 31% trainees, *P* = 0.002). If stopped, 41% cited opting for a drug holiday with monitoring. Forty percent of GPs gave patients a reminder card for the next injection and 27% had an alert system.

**Conclusion:**

We identified a knowledge gap in denosumab prescribing among a sample of Irish GPs. Findings suggest a need for education to increase awareness around denosumab use and to consider recall systems in GP practices as suggested elsewhere to ensure persistence with therapy.

## Introduction

Denosumab is commonly used by general practitioners (GPs) in Ireland in the treatment of osteoporosis [1]. It is a monoclonal antibody administered twice yearly as a subcutaneous injection and is often used in patients with contraindications to bisphosphonates including gastro-oesophageal reflux disease and renal impairment (eGFR < 30 ml/min) [1]. However, studies suggest that a significant proportion of Irish GPs use denosumab as a first line therapy which is generally recommended for bisphosphonates [2]. Importantly, unlike bisphoshonates where drug holidays are appropriate for some patients, they are not compatible with denosumab with the anti-resorptive effects wearing off after 6 months, resulting in rebound bone loss and risk of vertebral fractures [1]. In fact, all treatment gains in bone density can be lost within 12–24 months of stopping. For this reason, if denosumab is initiated in primary care, consultation with secondary care colleagues may be advisable given the need to have a long-term personalised osteoporosis management plan in place to enable denosumab to be stopped in a managed way, as necessary [3].

Guidelines have recently advised treatment with zoledronic acid if denosumab is stopped [3, 4]. Oral bisphosphonates could also be cautiously considered in some patients with a short duration of therapy (< 2.5 years) providing there is monitoring with bone turnover markers (BTM) [4]. However, access to these options is difficult for GPs and even with follow-up therapy, bone loss can occur in a significant proportion of patients [1, 5]. For this reason, the decision to prescribe denosumab needs to be carefully considered given that there is no data on safety and efficacy beyond 10 years [1]. Indeed, in patients at high risk of fracture, remaining on therapy indefinitely may be appropriate [6].

Finally, in routine clinical practice, GPs should not prescribe denosumab if there is hypocalcaemia and/or a vitamin D level below 50 nmol/l and also need to consider checking calcium levels post administration in high risk patients [7]. Clinical monitoring of serum calcium levels prior to every dose is recommended but is not mandatory and clinical judgement should be applied on an individual patient basis.

We aimed to ascertain GP knowledge and practice with regard to denosumab including (1) use in the last year, (2) use as a first line therapy and reasons for same, (3) duration of treatment, (4) monitoring of blood biochemistry and knowledge of recommendations for vitamin D status and calcium intake on treatment, (5) clinical staff administering the injection, (6) methods of recall for repeat injections and reliance on pharmacists for reminders, (7) knowledge of acceptable delay in receiving injections and perceived delays during COVID-19, (8) clinical practice if denosumab is stopped (after 2.5 years), as well as awareness of guidelines and if concerns about same and (9) percieved reasons for stopping denosumab.

## Methods

GP principals/trainers and GP trainees registered with the Irish College of General Practitioners were contacted by email and invited to complete an online anonymous questionnaire in January 2022 using SurveyMonkey (see Table 1). This comprised 25 questions detailing information on GP demographics/practice characteristics and on knowledge and clinical practice regarding denosumab therapy (see Table 1). Differences in categorical responses to questions by GPs and practice type were explored with ${\chi }^{2}$ test and signficance accepted when *P* < 0.05. Ethical approval was granted by the Irish College of General Practitioners (ICGP) ethics committee (Ref: ICGP_REC-2021-T26) (Table 2).

**Table 1:** GP questionnaire

**GP questionnaire**	
**1**	Please tick the box that represents your gender? (male/female/other)
**2**	Please tick the box that best represents the type of your work practice? (single, ≤ 3, ≥ 4)
**3**	Please describe your practice location (urban or rural)
**4**	Please indicate your current role (GP principal or trainor/GP registrar)
**5**	How many years of experience do you have working in GP? (< 5 yrs, 5–14 yrs, ≥ 15 yrs)
**6**	Have you prescribed denosumab for osteoporosis in the last year? Yes/No
**7**	Do you prescribe denosumab as a first line treatment for osteoporosis in primary care? Yes/No
**8**	If YES to previous question (Q7), Please choose the reason you prescribe it as first line therapy? (Convenience, renal impairment, poor compliance with oral therapy, severe osteoporosis)
**9**	How long do you prescribe denosumab for? (1–3 yrs, 3–5 yrs, 5–10 yrs, > 10 yrs, lifelong)
**10**	Do you check a patients serum calcium prior to denosumab? Yes/No
**11**	Do you check a patients serum calcium after denosumab? (routinely/patients at risk of hypocalcaemia)
**12**	Do you check a patients calcium intake before prescribing denosumab ? Yes/No
**13**	What is recommended daily intake of calcium for patients with osteoporosis? Open answer
**14**	What level of vitamin D [25(OH)D] should a patient have before receiving denosumab? (30–50 nmol/l, 50–75 nmmol/l, > 75 nmol/l, none of these)
**15**	Who routinely administers patients denosumab in your practice? (GP, nurse, both)
**16**	Do you give patients a reminder card of when their next dose of denosumab is due? Yes/No
**17**	Do you have an alert system in place to alert patients when their denosumab injection is due? Yes/No
**18**	If you have an alert system, can you comment on what you use? Open answer
**19**	How often do you rely on the patients’ pharmacist to remind them of when denosumab is due? (never, rarely or not often, some of the time, most of the time)
**20**	How long can you delay a patient’s 6 monthly denosumab dose? (Less than 7 months, more than 7 months)
**21**	How often was there a delay in patients receiving denosumab during the COVID-19 pandemic? (never, rarely or not often, some of the time, most of the time)
**22**	If denosumab is stopped after 2.5 years of treatment, what do you routinely do next?
**23**	Have you any concerns about a patient’s denosumab treatment being stopped? Yes/No
**24**	Are you aware of any recent guidelines on what to do after stopping denosumab therapy? Yes/No
**25**	In your opinion, what are the main reason(s) for patients stopping denosumab therapy? Open answer

**Table 2:** Questionnaire results

**Q**	**Question**	**Answers**	**%**
**1**	Gender	Male Female	67 33
**2**	GP practice size	Single 3 ≥ 4	10 35 55
**3**	GP location	Urban Rural	74 26
**4**	GP position	GP principal/trainor GP trainee	50 50
**5**	GP experience (years)	< 5 yrs 5–14 yrs ≥ 15 yrs	51 12 37
**6**	Denosumab used in the last year	Yes No	90 10
**7**	Denosumab used as a first line therapy	Yes No	43 57
**8**	Reason for use as a first line therapy *(*n* = 62)	GI contraindications Poor compliance Convenience Severe osteoporosis Renal impairment	49 42 32 14 7
**9**	Length of time denosumab prescribed	1–3 years 3–5 years 5–10 years > 10 years Lifelong	17 50 16 2 15
**10**	Serum calcium checked before starting denosumab	Yes No	79 21
**11**	Serum calcium checked after denosumab injection	Routinely Patients at risk	17 72
**12**	Calcium intake evaluated prior to starting therapy	Yes No	79 21
**13**	Knowledge of daily calcium intake *(*n* = 86)	≥ 1000 mg/day	54
**14**	25(OH)D level before starting denosumab	30–50 nmol/l 50–75 nmol/l > 75 nmol/l None of above	9 57 14 20
**15**	Staff rountinely administering denosumab	GP Nurse GP or Nurse	11 63 26
**16**	Use of reminder card for patients for next dose	Yes No	40 60
**17**	Alert system for patients for next dose	Yes	27
**18**	Type of alert system *(*n* = 27)	SMS text (*n* = 13) Phone call (*n* = 5) Email (*n* = 3) Nurse call (*n* = 3) Non specified (*n* = 3)	48 19 11 11 11
**19**	Reliance on pharmacist to remind patient of next dose	Never Rarely or not often Some of the time Most of the time	23 28 35 14
**20**	Knowledge of acceptable interval between injections	< 7 months > 7 months	63 37
**21**	Delay in receiving denosumab during COVID-19	Never Rarely or not often Some of the time Most of the time	3 26 60 11
**22**	Treatment if denosumab is stopped after 2.5 years	Drug holiday and monitor Unclear/refer elsewere Bisphosphonates/SERM	41 19 40
**23**	GP concerns if denosumab is stopped	Yes No	79 21
**24**	GP awareness of recent guidelines if stopping denosumab	Yes No	18 82
**25**	Main reason(s) for patients stopping denosumab	Concern about too long Noncompliance Side effects Perceived lack of benefit	43 32 29 14

Total number who answered survey was 146 except where indicated * (smaller sample size quoted). %, percentage calculated out of total survey sample except where smaller sample

*SERM* selective oestrogen receptor modulator

## Results

The survey was sent to 867 GPs and 17% (146) responded. Half (50%) comprised GP principals or trainers, the remainder GP trainees and two-thirds (67%) were female. The majority (74%) of practices were urban and 10% had one GP. Over a third (37%) were in a practice for more than 15 years, 12% for 5–14 years and 51% less than 5 years.

The vast majority (90%) had prescribed denosumab in the last year. Close to half (43%) had used it as a first line therapy with cited factors being gastrointestinal upset (49%), poor compliance with oral therapy (42%), convenience (32%), severe osteoporosis (14%) and renal impairment (7%). The most common time period envisaged for therapy duration was 3–5 years (50%) with 17% citing 1–3 years, 16% for 5–10 years, 2% more than 10 years and 15% lifelong use.

Over one in five (21%) responded that they did not check serum calcium prior to therapy and this was higher for GP registrars compared to trainers (26 vs 16%, *P* = 0.01). Approximately one in six (17%) cited checking serum calcium routinely after denosumab injections and 72% in patients at risk of hypocalcaemia. The minority (21%) did not assess total daily calcium intake and of 86 GPs who commented, 54% cited a recommended daily allowance in osteoporosis (≥ 1000 mg). Most (71%) considered that patients should have a vitamin D level of ≥ 50 nmol/l prior to starting denosumab, with 9% citing 30–50 nmol/l, 15% > 75 nmol/l and 20% none of these.

In most cases (63%), denosumab was administered by practice nurses only. Over one-third (40%) gave a reminder card for the next denosumab injection which was more likely in GP practices of ≥ 4 compared to 1–3 GPs (32 vs 27%, *P* = 0.01). Overall, 27% of GPs had alerts to remind patients of the need for repeat prescription. Systems included SMS text (13), phone call (5), email (3) and nurse call (3). Nearly half (49%) noted that they relied on pharmacists ‘some or most of the time’ to give reminders for a repeat prescription.

Over one-third (37%) were not aware that denosumab should not be delayed by > 7 months from the last injection with this being more likely in GP registrars compared to GP trainers (45 vs 29%, *P* = 0.04). The majority (71%) felt there was a delay in denosumab administration some or most of the time during COVID-19. There was no difference in the response by GP status (registrar vs trainer) as what to do if stopping denosumab after 2.5 years: 19% were unclear and might refer for specialist opinion, 41% cited a drug holiday and monitoring with DXA and about a third (38%) noted that an oral bisphosphonate or SERM should be started. Overall, 21% had no concerns about denosumab being stopped but this varied significantly from 11% in trainers to 31% in registrars, *P* = 0.002. About one in five (18%) were aware of any recent guidelines on what do if stopping denosumab.

The main reason cited why GPs felt patients stopped denosumab was due to concern about being on treatment too long (43%), noncompliance (32%), adverse effects (29%) and perceived lack of benefit (14%). Individual comments included that ‘dementia patients family may forget to bring their relative to the practice’ and that ‘some patients become housebound and current resources do not support house calls for administration’. One GP commented that they do not use denosumab anymore as ‘it is too risky’.

## Discussion

To our knowledge, this is the only study in Ireland that has explored both GP knowledge and clinical practice with regard to denosumab in the treatment of osteoporosis. We identified a knowledge gap in a number of areas, especially with regard to stopping treatment and follow-up therapy.

While the majority of GPs had used denosumab in the preceding year, close to half had prescribed it as first a first line treatment. In a recent study of 1146 Irish patients prescribed denosumab by GPs between 2012 and 2017, over half had no prior bone therapy suggesting a rate of first line use not explained by contraindications to other therapies [2]. We found that in a third of the cases, ‘convenience’ was cited as an indication for first line use; however, no other Irish studies have explored the reasons for denosumab prescribing. Other studies elsewhere have shown that patients have a preference for a six monthly injection compared to weekly tablets, with convenience being identified as an important factor [8]. This is likely to impact on GPs who take patient preferences into consideration when prescribing osteoporosis medications [9].

About half of GPs envisaged therapy duration for 3–5 years with practice nurses administering the injection in most cases. However, it is unclear how this compares to practice elsewhere or whether the availability of nurses to administer injections could influence GPs prescribing of denosumab. A minority did not check serum calcium prior to the injection though while assessing serum calcium is recommended before drug administration it is not mandatory. Indeed, during COVID-19, some guidelines waivered the advice to check serum calcium in all patients if normal in the previous year due to difficulties with accessing bloods and advised clinical judgement on an individudal basis [10]. On the other hand, about one in six checked calcium routinely post injection which is not necessary. Indeed, at the end of denosumab therapy (i.e. at about 6 months or more after the last injection), there may also be a mild hypercalcaemia associated with rebound phenomenon [11] that could inadvertently lead to delay in the next injection. However, importantly, the majority of GPs did check serum calcium post injection in patients at risk of hypocalcaemia. Just over half of GPs cited a daily calcium intake of 1000 mg or more for patients with osteoporosis. By comparison, in a survey of GP knowledge of osteoporosis in the Czech Republic in 2017, 41% were reported to correctly state the recommended calcium intake [12].

Most GPs had no alert systems to remind patients of their next dose and about half cited relying on pharmacists. While persistence with denosumab in Irish patients has been found to be 57% at 2 years, it has been reported to be higher in those with a medical card [2]. Medical card holders in Ireland are entitled to medications at no cost, with reimbursement of their dispensing pharmacist by the Irish Department of Health. Pharmacy oversight of these prescriptions might contribute to this better persistence though avoidance of an ‘out of pocket’ expense is also likely to be an important factor.

A third were not aware of the need for timely denosumab administration (no longer than 7 months after the last injection) suggesting a lack of knowledge among some GPs of current guidelines. This was also more likely in GP registrars who might be less aware of recommendations. Perhaps not surprisingly, the majority of GPs felt there was a delay in denosumab injections during COVID-19 as has been reported elsewhere [13]. Interestingly, the paradigm of drug holiday was considered by 41% if stopping denosumab despite the vast majority having concerns if there was therapy cessation. However, a significant proportion were unclear as to what to do if stopping and might refer for specialist opinion. By comparison, in a recently published Australian study, GPs expressed uncertainty about when to stop denosumab, what to do when stopping, the risk of stopping without an alternative being prescribed, or what should be prescribed if a patient had previously had problems with bisphosphonates [9].

Consistent with the above, we found that the majority of GPs had no knowledge of recent guidelines on what do if denosumab is stopped with just over one-third citing the use of an antiresorptive therapy after cessation. A previous study in Ireland found that 6% of patients who stopped denosumab were started on alternative treatments by their GP [2] while in Australia, this was reported to be less than 20% [9]. However, both studies reported on GP practices at a time when knowledge of the phenomenon of rebound bone loss on denosumab cessation was only emerging [1]. GPs felt that the commonest reason for patients wanting to stop denosumab was concern about being on treatment too long which is a similar to what as been identifed for other osteoporosis drugs [14].

We acknowledge that only 17% of GPs contacted replied to our survey which could bias the findings. However, the response rate to Irish GP surveys has been identified to be similarily low in other studies with the same methodology [15–18]. The quality or representativeness of a survey also does not necessarily correlate with its size, and a lower response rate does not necessarily make a survey less accurate [19]. Furthermore, previous research suggests that GPs with less interest in a topic may be less likely to engage in surveys [20, 21]. Therefore, this survey could potentially underestimate the knowledge gap identified.

In conclusion, we identified a knowledge gap with regard to denosumab prescribing among a sample of Irish GPs, particularly with regard to cessation of therapy and follow-up treatments. Our findings suggest that there is a need for education to increase awareness around denosumab use. It also highlghts the need for reminder or recall systems in GP practices so as to avoid rebound fractures [9].
